# Supplementary material for: Deep learning model for personalized prediction of positive MRSA culture using time-series electronic health records
Source: Nat Commun. 2024 Mar 6;15:2036. doi: 10.1038/s41467-024-46211-0 (PMC10917736; doi:10.1038/s41467-024-46211-0)
Supplement: Supplementary file 1 — Supplementary Information [file 41467_2024_46211_MOESM1_ESM.pdf]

Supplementary Materials

1. Supplementary Table 1: Features Included in This Project
2. Supplementary Table 2: Definition of MRSA Specific and Any MRSA Antibiotics in This Project
3. Supplementary Figure 1: PyTorch_EHR Pipeline and Processing Flow
4. Supplementary Figure 2: Modified Data Structure of PyTorch_EHR
5. Supplementary Figure 3: Data Structure for LR and LGBM
6. Supplementary Figure 4: Number of Inputs Available in Each Dataset
7. Supplementary Figure 5: Area Under Curve of Receiver Operating Characteristics
8. Supplementary Figure 6: Calibration Curve of PyTorch_EHR Model on MHHS Data
9. Supplementary Figure 7: Top 14 Admission Diagnosis that Model Provided High Contribution to Predict MRSA Positive Culture in MHHS Data
10. Supplementary Figure 8: Means of Feature Importance of Antimicrobials Exposed Before Index Time in MHHS and MIMIC-IV Data
11. Supplementary Figure 9: Features Contributing to the Individual Prediction in an Example Patient
12. Supplementary Table 3: Subgroup Analyses Results by PyTorch_EHR, Logistic Regression, and Light Gradient Boost Machine in MHHS and MIMIC-IV data
13. Supplementary Table 4: Confusion Matrices of all models on MHHS and MIMIC-IV Datasets
14. Supplementary Table 5: Potential Clinical Impart of Each Model
15. Supplementary Figure 10: AUROC Changes for Repeated Index Events

Supplementary Table 1: Features Included in This Project

Various tables/categories of structured electronic health records (EHR) data were used in this project. This table summarizes the features included.

**Category of Features**	**Features**
Demographics	Age[a], Gender, Ethnicity, Race, Language
Encounter Information	Encounter location (ICU, ED, IMU), Admission diagnosis (String, Not available in MIMIC-IV), Duration of admission/encounter (if discharged before index time)
Diagnostic code	ICD-10 or ICD-9 codes
Procedure code	Procedure code (CPT codes)
Antimicrobials	Antibiotic name (string), route of the antibiotic, IV access
ID-related test	HIV test, MRSA PCR, Legionella urine antigen, Treponemal antibody, etc. (150 different tests)
Cultures	Culture order, such as Blood culture, Culture source
Culture results	When the results were known before index time:<br>Positive, Negative, Isolate name
Sensitivity results	When the results were known before index time:<br>Name of bacteria, Name of tested antibiotic, Sensitivity interpretation of tested antibiotic against the isolate

ICD: International Classification of Disease, ICU: Intensive Care Unit, ID: Infectious Diseases, CPT: Current Procedural Terminology, ED: Emergency Department, IMU: Intermediate Unit, HIV: Human Immunodeficiency Virus, MRSA: Methicillin-Resistant *Staphylococcus aureus*.

a. Age (numerical) was converted to nominal data, such as age between 55 – 65 years old.

Supplementary Table 2: Definition of MRSA Specific and Any MRSA Antibiotics in This Project

Antibiotic Category	Included Antimicrobials
MRSA Specific Antibiotics	Vancomycin (Only IV), Daptomycin, Telavancin, Linezolid, Tedizolid
Any MRSA Antibiotics	Sulfamethoxazole-Trimethoprim, Minocycline, Doxycycline, Tigecycline, Eravacycline, Clindamycin, Dalbavancin, Oritavancin, Quinupristin /Dalfopristin

MRSA specific antibiotics include those often used to target MRSA severe infections, including bacteremia. Any MRSA antibiotics are those which have MRSA activity but also often used to treat other types of bacteria or used as outpatient.

Supplementary Figure 1: PyTorch_EHR Pipeline and Processing Flow

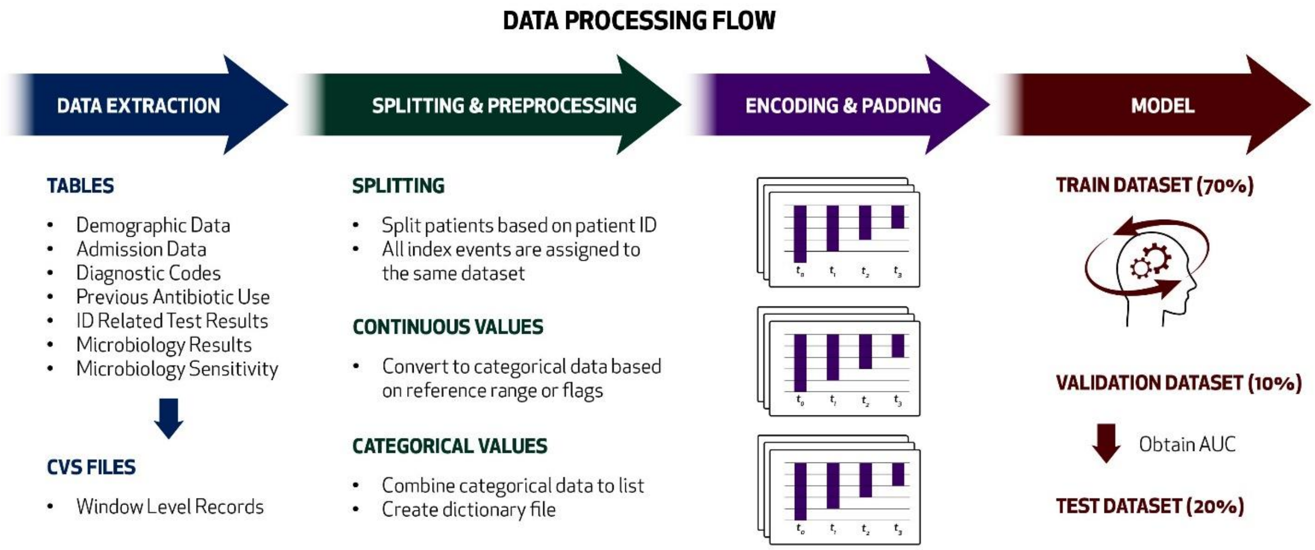

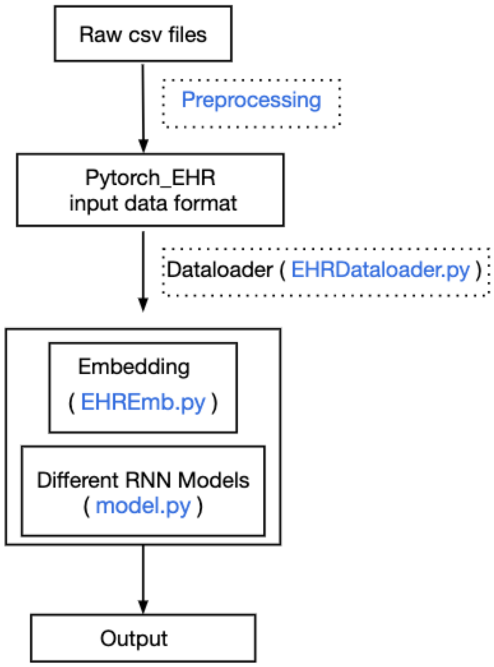

In this project, we used PyTorch_EHR scheme to predict positive MRSA cultures over two weeks from index culture. Original files were prepared as csv files for both Memorial Hermann Hospital System (MHHS) and Medical Information Mart for Intensive Care (MIMIC)-IV. Data were preprocessed to be compatible to data format for PyTorch_EHR. All data were used as categorical data. The features were embedded and tested on different recurrent neural network (RNN) and machine learning models.

Supplementary Figure 2-a: Modified Data Structure of PyTorch_EHR

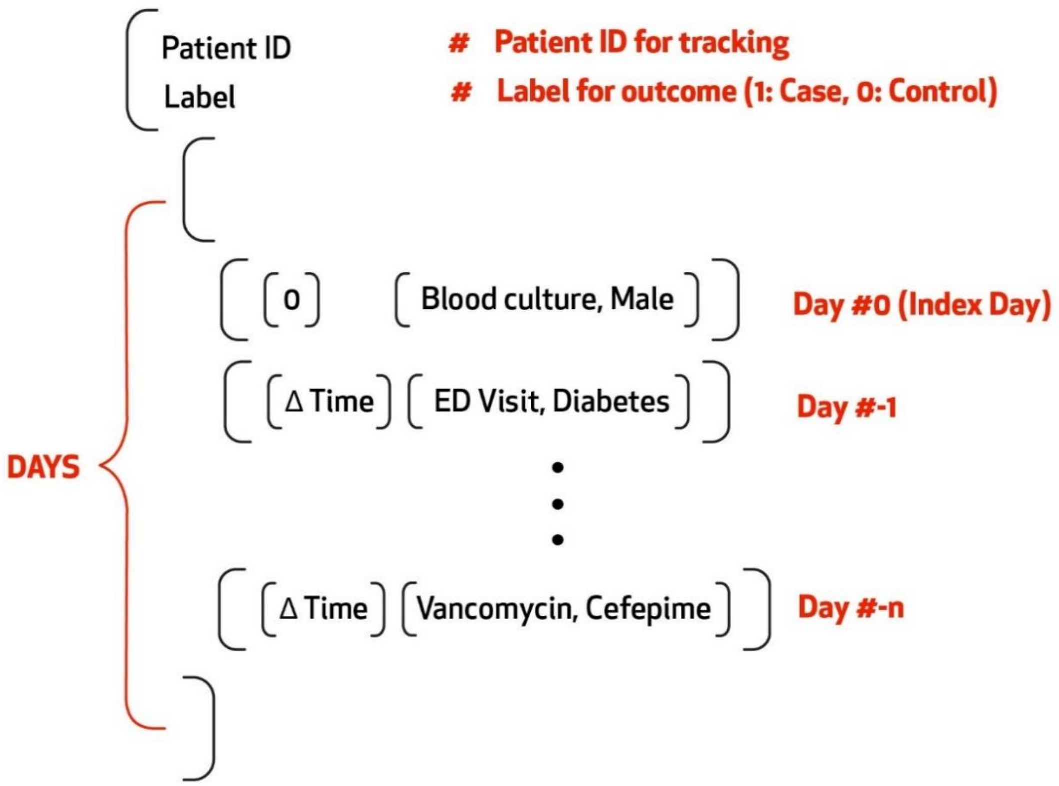

This is the data structure of PyTorch_EHR. Data for each patient have a patient ID and outcome label, followed by data inputs, including the delta time between the days of inputs and inputs features. Input features/clinical events can be multiple as any event happened on the day. Each feature was embedded before processing. During the training process, the model learns the relationship and timing of the features to predict the risk of positive MRSA cultures over two weeks.

Supplementary Figure 3: Data Structure for LR and LGBM

Patient_ID	Label	feature1	feature2	feature3	…	Elapsed time feature1	Elapsed time feature2	Elapsed time feature3	…
A	0	0	3	2	…	N/A	3	4	…
		⋮					⋮		
B	1	1	0	3	…	0	N/A	2	…
C	0	2	2	0		1	0	N/A	

The number of each feature represents how many times the features occurred in the datasets before the index time. The elapsed time feature is the time differences between index time and the most recent features occurred.

Supplementary Figure 4: Number of Inputs Available in Each Dataset

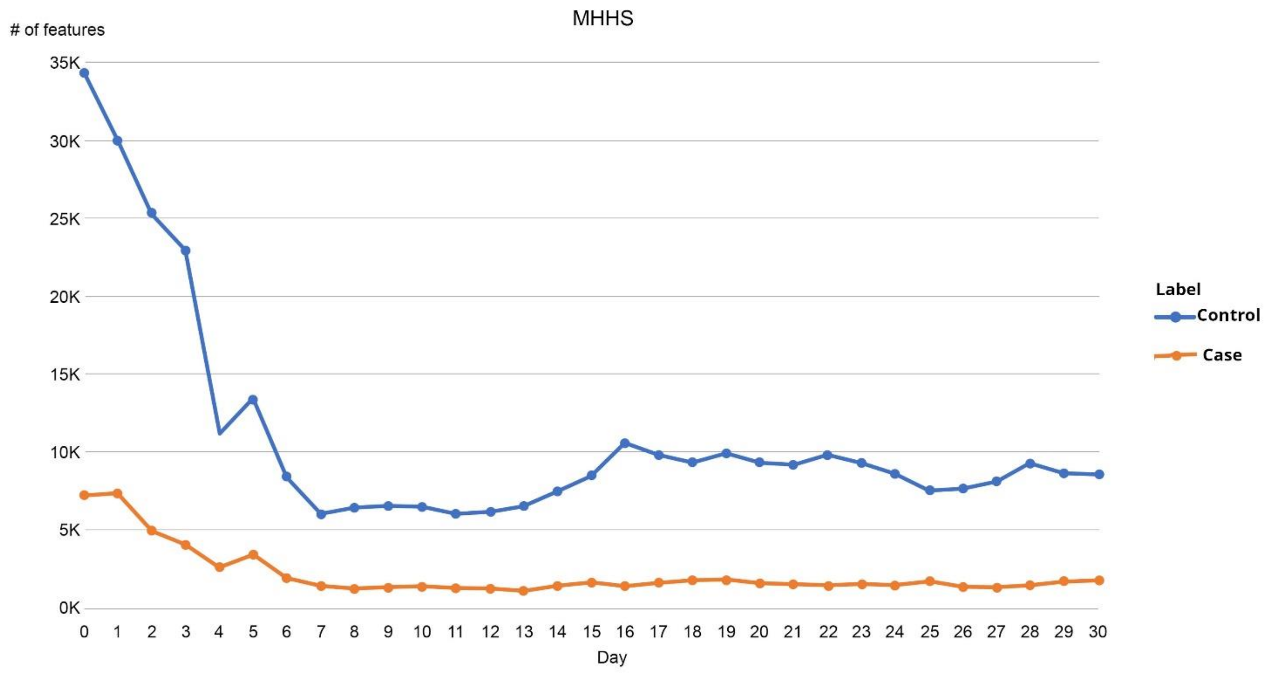

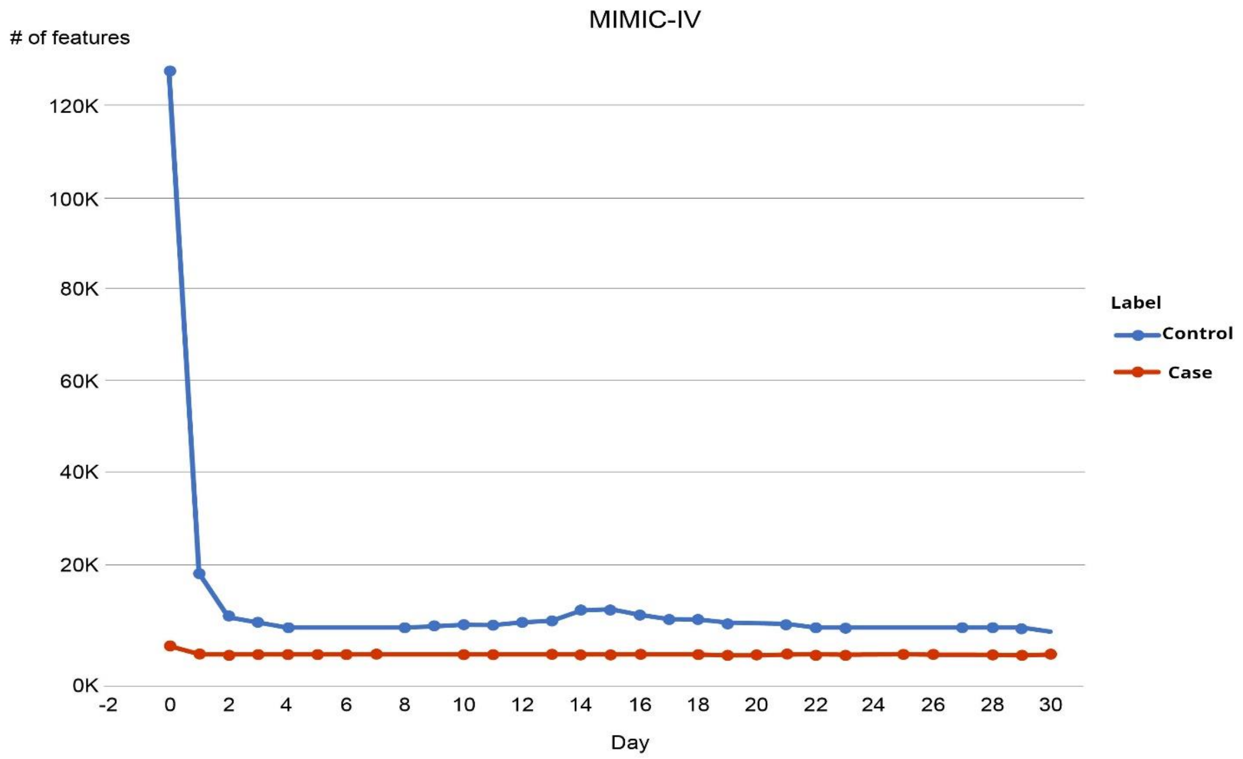

The x-axis represents the days before the index culture, and the y-axis represents the number of inputs on the day. MHHS patients had higher rates of inputs before the index date whereas MIMIC-IV patients had the majority of input data on the index date.

Supplementary Figure 5: Area Under Curve of Receiver Operating Characteristics

Supplementary Figure 5-1: MHHS Data

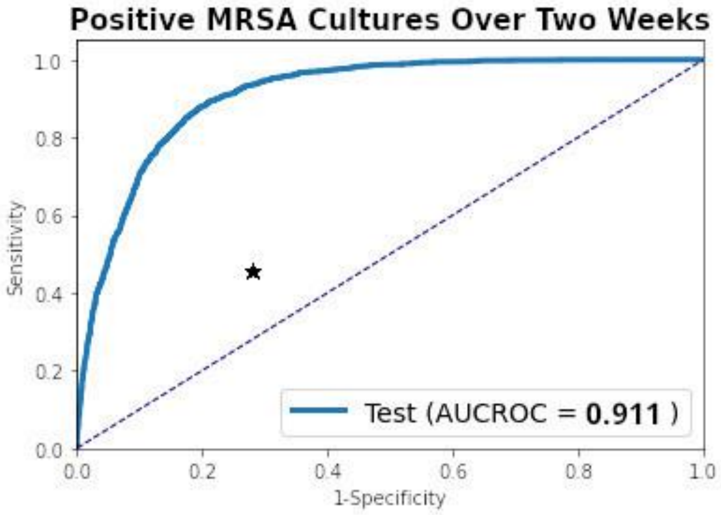

★Physician's prescriptions against MRSA cultures:

MRSA specific antimicrobials: Sensitivity 41.9%, Sensitivity: 72.8%

Any MRSA antimicrobials: Sensitivity 46.1%, Sensitivity: 69.8%

Supplementary Figure 5-2: MIMIC-IV Data

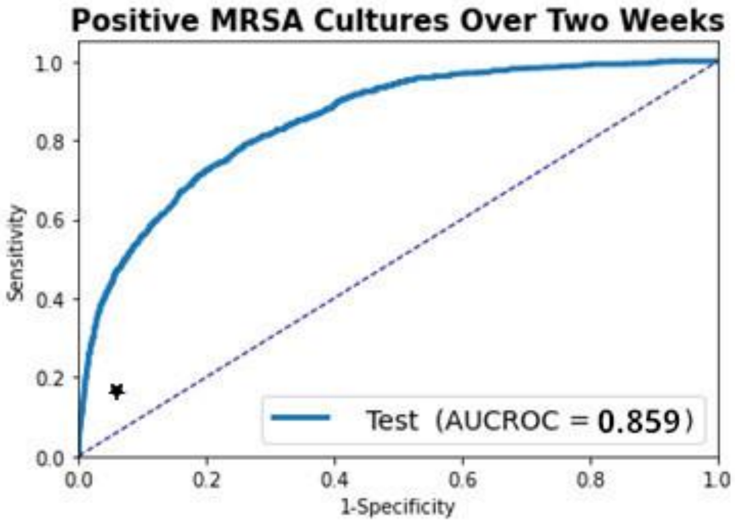

★Physician's prescriptions against MRSA cultures:

MRSA specific antimicrobials: Sensitivity 16.3%, Sensitivity: 95.4%

Any MRSA antimicrobials: Sensitivity 17.5%, Sensitivity: 94.4%

Supplementary Figure 6: Calibration Curve of each model for MHHS and MIMIC-IV datasets

Supplementary Figure 6-a PyTorch_EHR Model on MHHS Data

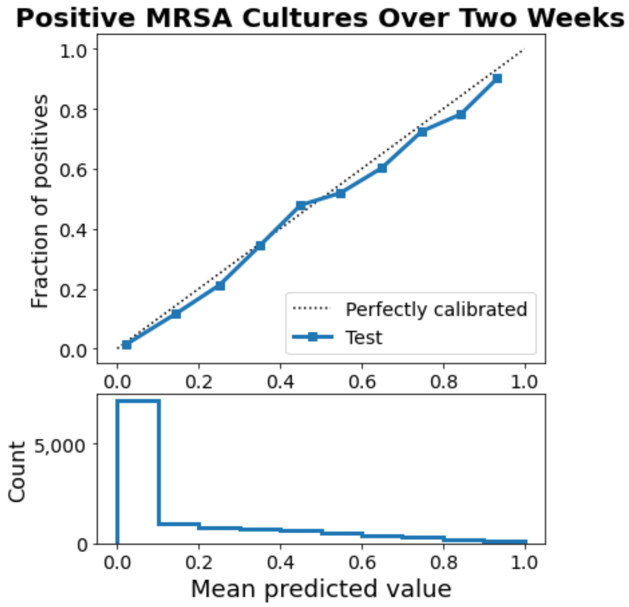

Supplementary Figure 6-b LGBM Model on MHHS Data

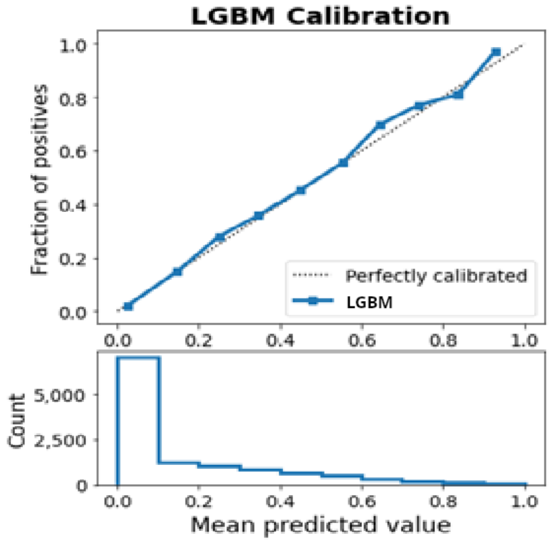

Brier Score: 0.09

Supplementary Figure 6-c LR Model on MHHS Data

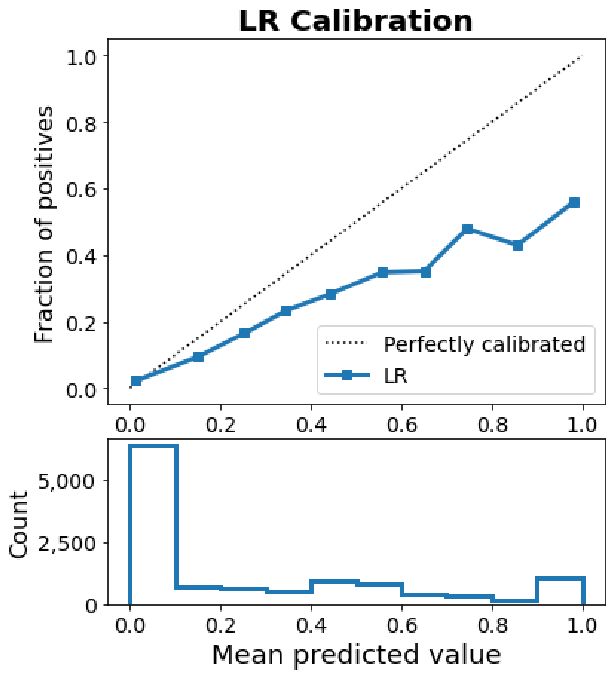

Brier Score: 0.13

Supplementary Figure 6-d PyTorch_EHR Pretrained Model on MIMIC-IV Data

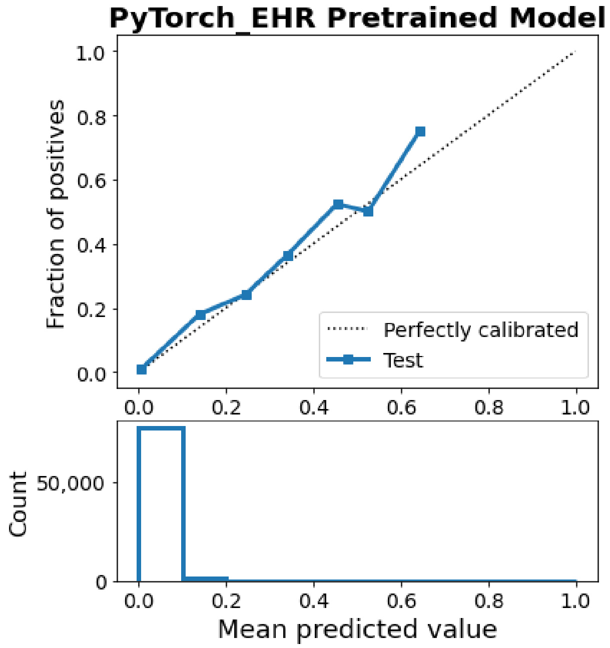

Brier Score: 0.012

Supplementary Figure 6-f LGBM Model on MIMIC-IV Data

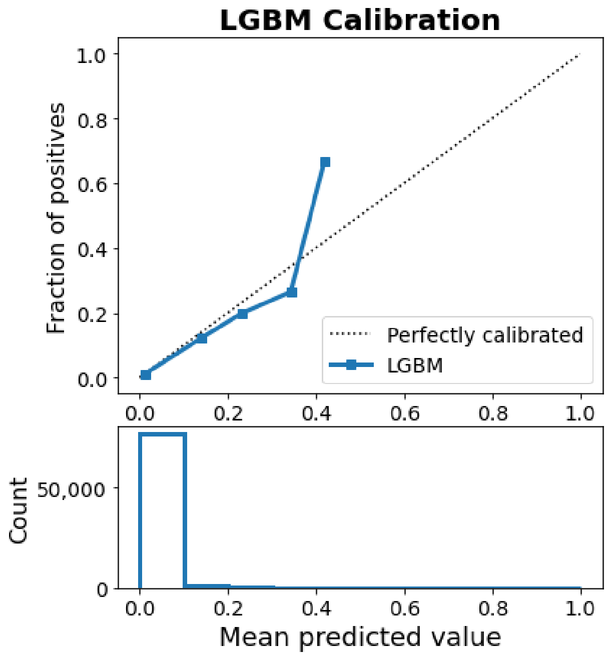

Brier Score: 0.013

Supplementary Figure 6-g LGBM Model on MIMIC-IV Data after calibration

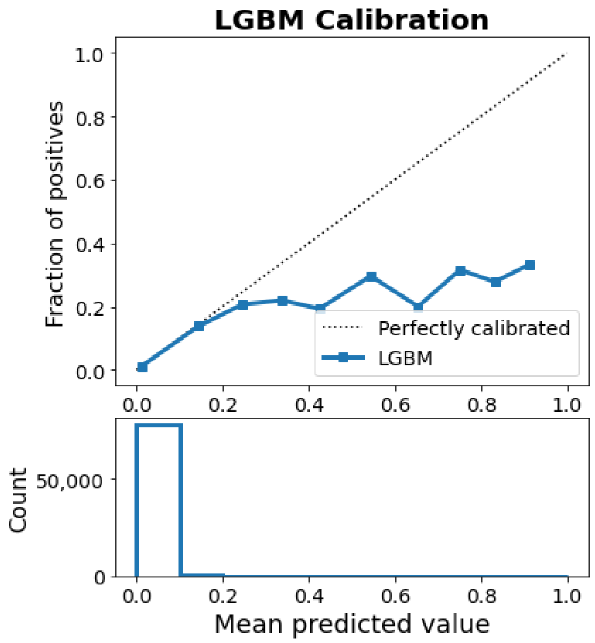

Brier Score: 0.013

Supplementary Figure 6-h LR Model on MIMIC-IV Data

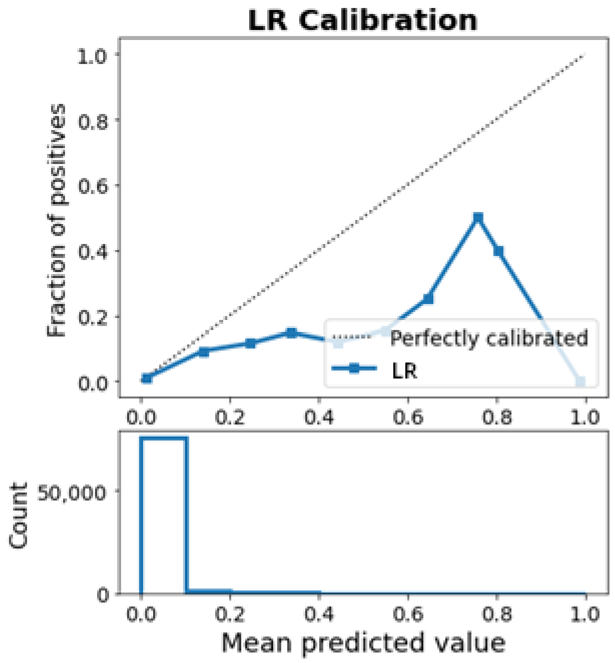

Brier Score: 0014

Supplementary Figure 7: Top 14 Admission Diagnosis that Model Provided High Contribution to Predict MRSA-Positive Culture in MHHS Data

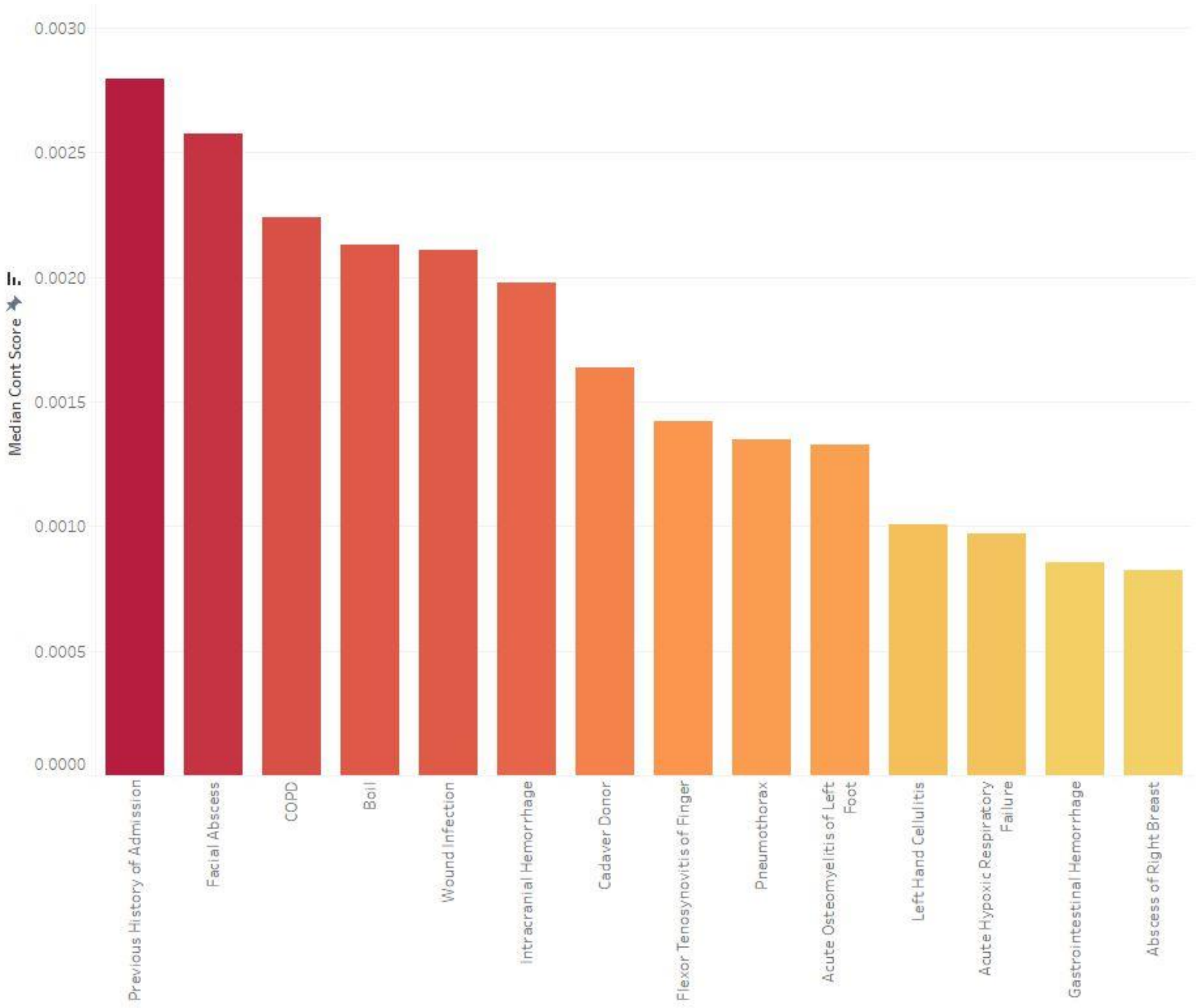

We obtained median contribution scores provided by PyTorch_EHR for admission diagnosis in MHHS data. A higher score means the admission diagnosis strongly contributed to provide positive MRSA culture prediction in the dataset. Although some of the diagnoses were not related to MRSA infections, multiple admission diagnoses such as soft tissue abscesses contributed to the positive prediction.

Supplementary Figure 8: Means of Feature Importance of Antimicrobials Exposed Before Index Time in MHHS and MIMIC-IV Data

MHHS Data

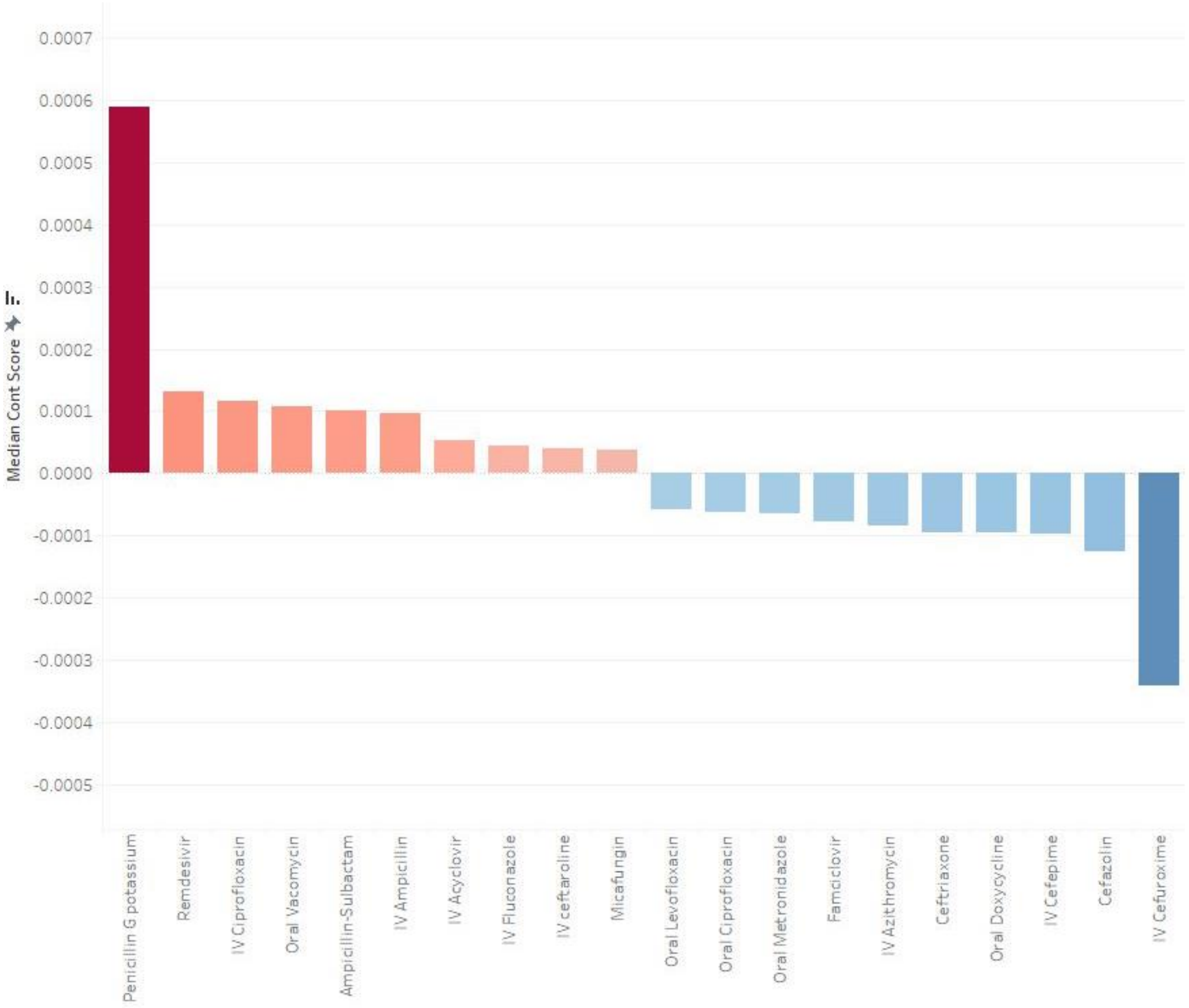

MIMIC-IV Data

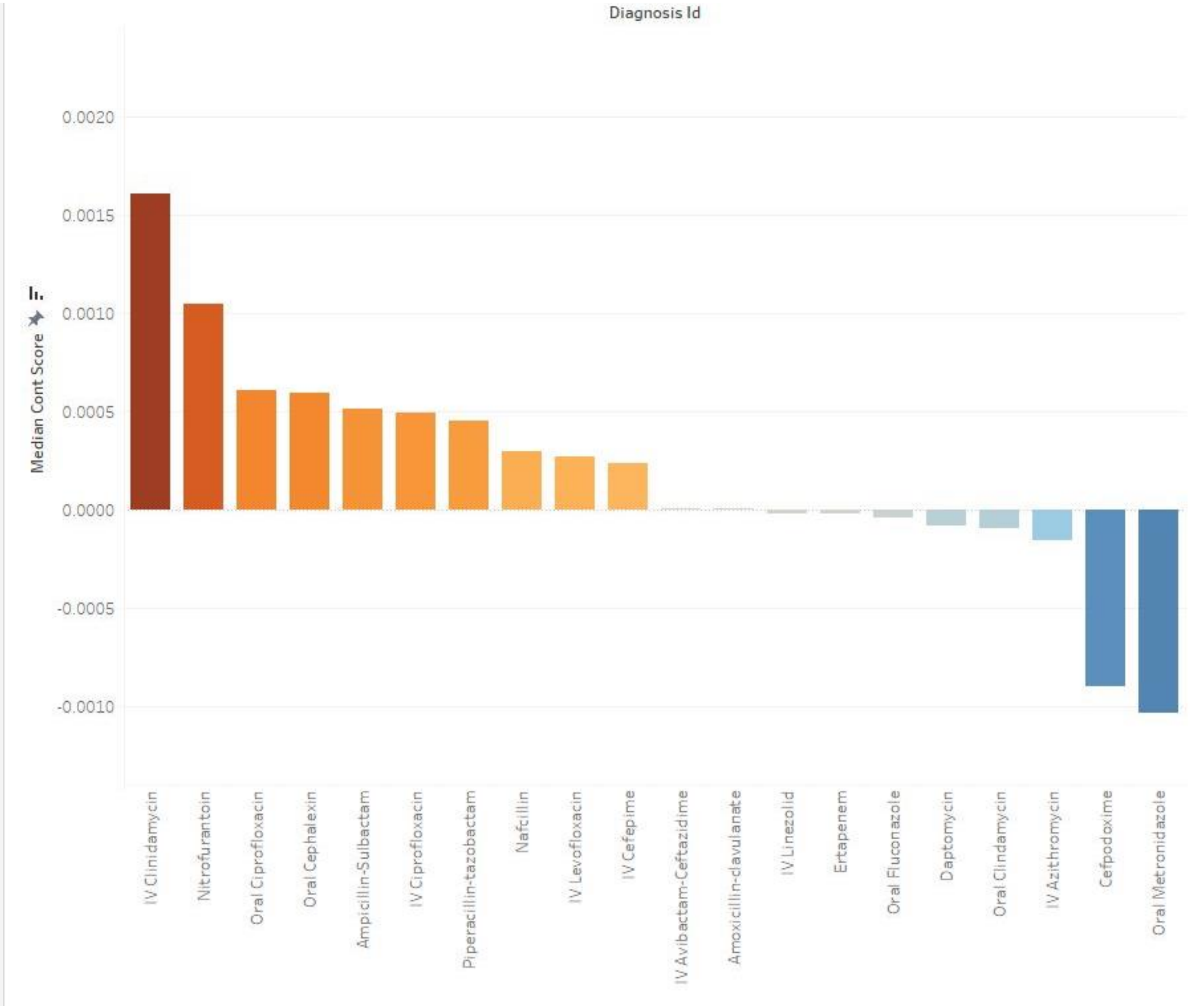

Supplementary Figure 9: Features Contributing to the Individual Prediction in an Example Patient

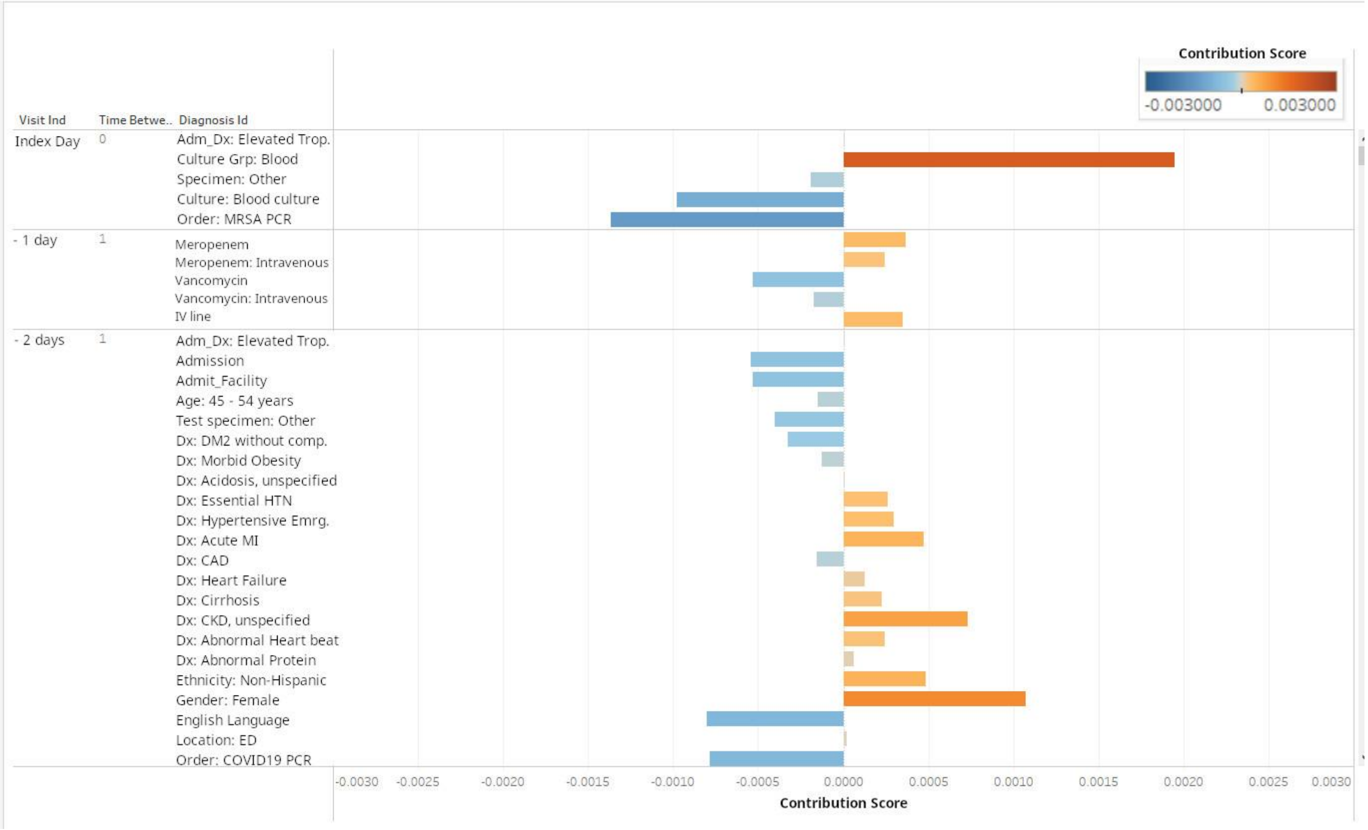

This patient was a female patient between 45 – 54 years of age with multiple underlying comorbidities listed at admission two days (-2 days) before the index culture (blood culture on index date). Our model identified the risk score as 0.541. After the patient was admitted to the hospital, she started vancomycin and meropenem, and a blood culture was ordered. Subsequently, cultures identified MRSA over two weeks.

Supplementary Table 3: Subgroup Analysis Results by PyTorch_EHR, Logistic Regression, and Light Gradient Boost Machine in MHHS and MIMIC-IV data

Subgroups Analysis		***MHHS*** ***Mean (CI)***	***MIMIC-IV*** ***Mean (CI)***
Sepsis	**LR**	0.733 (0.706 – 0.759)	0.690 (0.636 – 0.744)
	**LGBM**	0.829 (0.808 – 0.849)	0.743 (0.692 – 0.794)
	**PyTorch_EHR**	**0.864 (0.846 – 0.882)***	0.789 (0.740 – 0.838)
	**PyTorch_EHR Pre-Trained**	-	0.781 (0.734 – 0.828)
Bacteremia	**LR**	0.686 (0.828 – 0.743)	0.700 (0.648 – 0.752)
	**LGBM**	0.853 (0.815 – 0.891)	0.758 (0.712 – 0.805)
	**PyTorch_EHR**	0.879 (0.842 – 0.915)	0.797 (0.755 – 0.840)
	**PyTorch_EHR Pre-Trained**	-	0.809 (0.770 – 0.848)
Pneumonia	**LR**	0.741 (0.706 – 0.775)	0.706 (0.661 – 0.751)
	**LGBM**	0.836 (0.810 – 0.862)	0.756 (0.715 – 0.797)
	**PyTorch_EHR**	**0.872 (0.849 – 0.894)***	0.783 (0.743 – 0.823)
	**PyTorch_EHR Pre-Trained**	-	0.769 (0.730 – 0.807)
Skin Soft Tissue Infections	**LR**	0.711 (0.679 – 0.815)	0.761 (0.721 – 0.802)
	**LGBM**	0.788 (0.760 – 0.815)	0.769 (0.727 – 0.810)
	**PyTorch_EHR**	0.804 (0.778 – 0.831)	0.819 (0.783 – 0.856)
	**PyTorch_EHR Pre-Trained**	-	0.811 (0.775 – 0.847)

LR: Logistic Regression, LGBM: Light Gradient Boost Machine

* AUC of PyTorch_EHR was significantly higher than one of LGBM

Supplementary Table 4: Confusion Matrices of PyTorch_EHR on MHHS and MIMIC-IV Datasets

MHHS Data

High Risk: Pytorch_EHR

Prediction	MRSA Cx+	MRSA Cx-
Positive	943	494
Negative	1017	9468

**Sensitivity**	48.1%
**Specificity**	95.0%
**PPV**	65.6%
**NPV**	90.3%

High Risk: LGBM

Prediction	MRSA Cx+	MRSA Cx-
Positive	872	498
Negative	1088	9464

**Sensitivity**	44.5%
**Specificity**	95.0%
**PPV**	63.6%
**NPV**	89.7%

High Risk: LR

Prediction	MRSA Cx+	MRSA Cx-
Positive	622	495
Negative	1338	9467

**Sensitivity**	31.8%
**Specificity**	95.0%
**PPV**	55.7%
**NPV**	87.6%

Low Risk: Pytorch_EHR

Prediction	MRSA Cx+	MRSA Cx-
Positive	1862	3085
Negative	98	6877

**Sensitivity**	95.0%
**Specificity**	62.9%
**PPV**	37.6%
**NPV**	98.6%

Low Risk: LGBM

Prediction	MRSA Cx+	MRSA Cx-
Positive	1862	3697
Negative	98	6265

**Sensitivity**	95.0%
**Specificity**	62.8%
**PPV**	33.5%
**NPV**	98.5%

Low Risk: LR

Prediction	MRSA Cx+	MRSA Cx-
Positive	1862	5625
Negative	98	4337

Sensitivity	95.0%
Specificity	56.5%
PPV	30.0%
NPV	98.3%

MIMIC-IV Data

High Risk: PyTorch_EHR

Prediction	MRSA Cx+	MRSA Cx-
Positive	214	743
Negative	892	76699

Sensitivity	19.3%
Specificity	99.0%
PPV	22.4%
NPV	98.9%

High Risk: LGBM

Prediction	MRSA Cx+	MRSA Cx-
Positive	165	777
Negative	941	76665

Sensitivity	14.9%
Specificity	99.0%
PPV	17.5%
NPV	98.8%

High Risk: LR

Prediction	MRSA Cx+	MRSA Cx-
Positive	130	751
Negative	976	76691

Sensitivity	11.8%
Specificity	99.0%
PPV	14.8%
NPV	98.7%

Low Risk: PyTorch_EHR

Prediction	MRSA Cx+	MRSA Cx-
Positive	998	32017
Negative	108	45425

Sensitivity	90.0%
Specificity	58.7%
PPV	3.0%
NPV	99.8%

Low Risk: LGBM

Sensitivity	90.0%

Prediction	MRSA Cx+	MRSA Cx-
Positive	995	33116
Negative	111	44326

Specificity	57.2%
PPV	2.9%
NPV	99.8%

Low Risk: LR

Prediction	MRSA Cx+	MRSA Cx-
Positive	994	35667
Negative	112	41775

Sensitivity	90.0%
Specificity	53.9%
PPV	2.7%
NPV	99.7%

Cx: Culture, LGBM: Light Gradient Boost Machine, LR: Logistic Regression, MRSA: Methicillin Resistant *Staphylococcus aureus*, NPV: Negative Predictive Value, PPV: Positive Predictive Value

Supplementary Table 5: Potential Clinical Impart of Each Model

These tables summarize the potential clinical benefits or harms of each model compared to the treating clinician's decisions. The numbers are obtained based on the antimicrobials possesses MRSA specific antimicrobials and any MRSA activities. For MRSA bacteremia, we only evaluated MRSA specific antimicrobials as other antimicrobials could be inappropriate against MRSA bacteremia.

Any MRSA antimicrobial analyses may underestimate the benefit of our model in early initiation of MRSA targeted empirical therapy given some of the antimicrobials are not appropriate for severe infections, such as MRSA bacteremia. Unnecessary antimicrobials may be overestimated as those were used for other purposes, such as gram-negative bacteria. Shaded bold rows are potential benefits from our model, and shaded italic rows are potential harms with our model. In each dataset, overall net benefit outweighed the harms from our model even compared to clinician's decision.

Supplementary Table 5-a: Potential Clinical Impact of PyTorch_EHR Model Based on MRSA Specific Antimicrobials

MHHS Data (PyTorch_EHR)						
**Model Predictions**		**MRSA Cx**	**Treating Clinician's decision**	**Cases**	**Potential Benefit and Harm of Model**	**Overall Potential Benefit**
**High Risk 1,437 PPV: 65.6**	**True Positive**	+	Empirically Treat	446/1,437(31.0%)	NA	**474 cases may receive early MRSA Abx**
	**943(65.6%)**	**+**	**Not Empirically Treat**	**497/1,437 (34.6%)**	**497 cases may get early MRSA Abx.**	
	**False Positive**	-	Empirically Treat	267/1,437 (18.5%)	NA	
	**494 (35.4%)**	*-*	*Not Empirically Treat*	*227/1,437 (15.8%)*	*227 cases may receive unnecessary MRSA Abx.*	
**Low Risk 6,975 NPV: 98.6**	**True Negative**	**-**	**Empirically Treat**	**1,505/6,975 (21.6%)**	**1,505 cases may avoid unnecessary MRSA Abx.**	**1278 cases may avoid unnecessary MRSA Abx**
	**6,877 (98.7%)**	-	Not Empirically Treat	5,372/6,975 (77.0%)	NA	
	**False Negative**	*+*	*Empirically Treat*	*23/6,975 (0.3%)*	*23 cases may delay MRSA Abx*	
	**98 (1.4%)**	+	Not Empirically Treat	75/6,975 (1.1%)	NA	
**MRSA Bacteremia 457**	**True Positive**	+	Empirically Treat	169/457 (37.0%)	NA	**250 MRSA bacteremia cases may start early MRSA Abx**
	**434 (95.0%)**	**+**	**Not Empirically Treat**	**265/457 (58.0%)**	**265 cases may start early MRSA Abx**	
	**False Negative**	*+*	*Empirically Treat*	*15/457 (3.3%)*	*15 cases may dealy MRSA Abx*	
	**23 (5.0%)**	+	Not Empirically Treat	17/457 (3.7%)	NA	
**MIMIC-IV (PyTorch_EHR)**						
**Model Predictions**		**MRSA Cx**	**Treating Clinician's decision**	**Cases**	**Potential Benefit and Harm of Model**	**Overall Potential Benefit**
**High Risk 957 PPV: 22.4**	**True Positive**	+	Empirically Treat	25/957 (2.6%)	NA	**162 cases may receive early MRSA Abx**
	**214 (21.1%)**	**+**	**Not Empirically Treat**	**189/957 (19.7%)**	**189 cases may get early MRSA Abx**	
	**False Positive**	-	Empirically Treat	72/957 (7.5%)	NA	
	**743 (77.6%)**	*-*	*Not Empirically Treat*	*671/957 (70.1%)*	*671 cases may receive unnecessary MRSA Abx*	
**Low Risk 45,533 NPV: 99.8**	**True Negative**	**-**	**Empirically Treat**	**1,069/45,533 (2.3%)**	**1,069 cases may avoid unnecessary MRSA Abx**	**398 cases may avoid unnecessary MRSA Abx**
	**45,425 (99.8%)**	-	Not Empirically Treat	44,356/45,533 (97.4%)	NA	
	**False Negative**	*+*	*Empirically Treat*	*27/45,533 (0.04%)*	*27 cases may delay MRSA Abx*	
	**108 (0.2%)**	+	Not Empirically Treat	81/45,533 (0.17%)	NA	
**MRSA Bacteremia 70**	**True Positive**	+	Empirically Treat	8/70 (11.4%)	NA	**10 MRSA bacteremia cases may receive early MRSA Abx**
	**35 (50.0%)**	**+**	**Not Empirically Treat**	**27/70 (38.6%)**	**27 cases may start early MRSA Abx**	
	**False Negative**	*+*	*Empirically Treat*	*17/70 (24.3%)*	*17 cases may dealy MRSA Abx*	
	**35 (50.0%)**	+	Not Empirically Treat	18/70 (25.7%)	NA	

Supplementary Table 5-b: Potential Clinical Impact of LGBM Model Based on MRSA Specific Antimicrobials

MHHS Data (LGBM)						
**Model Predictions**		**MRSA Cx**	**reating Clinician's decisio**	**Cases**	**Potential Benefit and Harm of Model**	**Overall Potential Benefit**
**High Risk 1,370 PPV: 63.6**	**True Positive 872 (63.9%)**	+	Empirically Treat	365/1370 (26.6%)	NA	**486 cases may receive early MRSA Abx**
		**+**	**Not Empirically Treat**	**507/1370 (37.0%)**	**507 cases may get early MRSA Abx.**	
	**False Positive 498 (36.4%)**	-	Empirically Treat	236/1370 (17.2%)	NA	
		**-**	*Not Empirically Treat*	*262/1370 (19.2%)*	*265 cases may receive unnecessary MRSA Abx.*	
**Low Risk 6,363 NPV:98.5**	**True Negative 6,265 (98.5%)**	**-**	**Empirically Treat**	**1,148/6,363 (18.5%)**	**1,148 cases may avoid unnecessary MRSA Abx.**	**883 cases may avoid unnecessary MRSA Abx**
		-	Not Empirically Treat	5,117/6,363 (80.4%)	NA	
	**False Negative 98 (1.5%)**	+	*Empirically Treat*	*21/6,363 (0.33%)*	*21 cases may delay MRSA Abx*	
		+	Not Empirically Treat	77/6,363 (1.2%)	NA	
**MRSA Bacteremia 393**	**True Positive 370 (80.8%)**	+	Empirically Treat	152/393 (38.7%)	NA	**198 MRSA bacteremia cases may start early MRSA Abx**
		**+**	**Not Empirically Treat**	**218/393 (55.4.%)**	**218 cases may start early MRSA Abx**	
	**False Negative 23 (19.2%)**	+	*Empirically Treat*	*20/393 (5.1%)*	*20 cases may dealy MRSA Abx*	
		+	Not Empirically Treat	3/393 (0.8%)	NA	
**MIMIC-IV (LGBM)**						
**Model Predictions**		**MRSA Cx**	**reating Clinician's decisio**	**Cases**	**Potential Benefit and Harm of Model**	**Overall Potential Benefit**
**High Risk 942 PPV: 17.5**	**True Positive 165 (17.5%)**	+	Empirically Treat	17/942 (1.8%)	NA	**129 cases may receive early MRSA Abx**
		**+**	**Not Empirically Treat**	**148/942 (15.7%)**	**148 cases may get early MRSA Abx**	
	**False Positive 777 (82.5%)**	-	Empirically Treat	58/942 (6.2%)	NA	
		-	*Not Empirically Treat*	*719/942 (76.3%)*	*719 cases may receive unnecessary MRSA Abx*	
**Low Risk 44,437 NPV: 99.8**	**True Negative 48,343 (99.8%)**	**-**	**Empirically Treat**	**922/44,437 (2.1%)**	**910 cases may avoid unnecessary MRSA Abx**	**191 cases may avoid unnecessary MRSA Abx**
		-	Not Empirically Treat	43,404/44,437 (97.7%)	NA	
	**False Negative 112 (0.2%)**	+	*Empirically Treat*	*19/44,437 (0.04%)*	*19 cases may delay MRSA Abx*	
		+	Not Empirically Treat	92/44,437 (0.2%)	NA	
**MRSA Bacteremia 85**	**True Positive 47 (55.3%)**	+	Empirically Treat	7/85 (8.2%)	NA	**18 MRSA bacteremia cases may receive early MRSA Abx**
		**+**	**Not Empirically Treat**	**40/85 (47.1%)**	**40 cases may start early MRSA Abx**	
	**False Negative 38 (44.7%)**	+	*Empirically Treat*	*22/85 (25.9%)*	*22 cases may dealy MRSA Abx*	
		+	Not Empirically Treat	16/85 (18.8%)	NA	

Supplementary Table 5-c: Potential Clinical Impact of LR Model Based on MRSA Specific Antimicrobials

MHHS Data (LR)						
**Model Predictions**		**MRSA Cx**	**Treating Clinician's decision**	**Cases**	**Potential Benefit and Harm of Model**	**Overall Potential Benefit**
**High Risk 1,117 PPV:55.7**	**True Positive 1,002 (64.7%)**	+	Empirically Treat	315/1,117 (28.2%)	NA	**292 cases may receive early MRSA Abx**
		**+**	**Not Empirically Treat**	**307/1,117 (27.5%)**	**307 cases may get early MRSA Abx.**	
	**False Positive 547 (35%)**	-	Empirically Treat	277/1,117 (24.8%)	NA	
		-	*Not Empirically Treat*	*218/1.117 (19.5%)*	*218 cases may receive unnecessary MRSA Abx.*	
**Low Risk 5,726 NPV:98.3**	**True Negative 5,628 (98.3%)**	**-**	**Empirically Treat**	**822/5,726 (14.4%)**	**822 cases may avoid unnecessary MRSA Abx.**	**604 cases may avoid unnecessary MRSA Abx**
		-	Not Empirically Treat	4,806/5,726 (71.5%)	NA	
	**False Negative 98 (0.7%)**	+	*Empirically Treat*	*15/5,726 (0.2%)*	*15 cases may delay MRSA Abx*	
		+	Not Empirically Treat	83/5,726 (1.4%)	NA	
**MRSA Bacteremia 336**	**True Positive 327 (97.3%)**	+	Empirically Treat	225/336 (37.0%)	NA	**102 MRSA bacteremia cases may start early MRSA Abx**
		**+**	**Not Empirically Treat**	**102/336 (30.4.%)**	**102 cases may start early MRSA Abx**	
	**False Negative 9 (2.7%)**	+	*Empirically Treat*	*0/336 (0%)*	*0 cases may dealy MRSA Abx*	
		+	Not Empirically Treat	9/336 (2.7%)	NA	
**MIMIC-IV (LR)**						
**Model Predictions**		**MRSA Cx**	**Treating Clinician's decision**	**Cases**	**Potential Benefit and Harm of Model**	**Overall Potential Benefit**
**High Risk 2,187 PPV:22.4**	**True Positive 261 (11.9%)**	+	Empirically Treat	34/2,187 (1.6%)	NA	**249 cases may receive early MRSA Abx**
		**+**	**Not Empirically Treat**	**227/2,187 (10.4%)**	**227 cases may get early MRSA Abx**	
	**False Positive 1926 (88.0%)**	-	Empirically Treat	177/2,187 (8.1%)	NA	
		-	*Not Empirically Treat*	*1,749/2,187 (80.0%)*	*1,749 cases may receive unnecessary MRSA Abx*	
**Low Risk 41,887 NPV:99.7**	**True Negative 41,775 (99.7%)**	**-**	**Empirically Treat**	**887/41,887 (2.1%)**	**887 cases may avoid unnecessary MRSA Abx**	*862 cases may receive unnecessary MRSA Abx*
		-	Not Empirically Treat	40,888/41,887 (97.7%)	NA	
	**False Negative 112 (0.3%)**	+	*Empirically Treat*	*22/41,887 (0.05%)*	*22 cases may delay MRSA Abx*	
		+	Not Empirically Treat	90/41,887 (0.2%)	NA	
**MRSA Bacteremia 85**	**True Positive 42 (49.4%)**	+	Empirically Treat	7/85 (8.2%)	NA	**15 MRSA bacteremia cases may receive early MRSA Abx**
		**+**	**Not Empirically Treat**	**35/85 (41.2%)**	**35 cases may start early MRSA Abx**	
	**False Negative 43 (50.6%)**	+	*Empirically Treat*	*20/85 (23.5%)*	*20 cases may dealy MRSA Abx*	
		+	Not Empirically Treat	23/85 (27.1%)	NA	

Supplementary Table 5-d: Potential Clinical Impact of PyTorch_EHR Model Based on Any Antimicrobials Possessing MRSA Activities

MHHS Data (PyTorch_EHR)						
**Model Predictions**		**MRSA Cx**	**Treating Clinician's decision**	**Cases**	**Potential Benefit and Harm of Model**	**Overall Potential Benefit**
**High Risk 1,437 PPV: 65.6**	**True Positive 943(65.6%)**	+	Empirically Treat	499/1,437(34.7%)	NA	**418 cases may receive early MRSA Abx**
		+	**Not Empirically Treat**	**444/1,437 (34.6%)**	**444 cases may get early MRSA Abx.**	
	**False Positive 494 (35.4%)**	-	Empirically Treat	282/1,437 (19.6%)	NA	
		-	*Not Empirically Treat*	*212/1,437 (14.8%)*	*212 cases may receive unnecessary MRSA Abx.*	
**Low Risk 6,975 NPV: 98.6**	**True Negative 6,877 (98.7%)**	-	**Empirically Treat**	**1,698/6,975 (24.3%)**	**1,698 cases may avoid unnecessary MRSA Abx.**	**1486 cases may avoid unnecessary MRSA Abx**
		-	Not Empirically Treat	5,179/6,975 (74.3%)	NA	
	**False Negative 98 (1.4%)**	+	*Empirically Treat*	*26/6,975 (0.4%)*	*26 cases may delay MRSA Abx*	
		+	Not Empirically Treat	72/6,975 (1.0%)	NA	
**MIMIC-IV (PyTorch_EHR)**						
**Model Predictions**		**MRSA Cx**	**Treating Clinician's decision**	**Cases**	**Potential Benefit and Harm of Model**	**Overall Potential Benefit**
**High Risk 957 PPV: 22.4**	**True Positive 214 (21.1%)**	+	Empirically Treat	27/957 (2.8%)	NA	**157 cases may receive early MRSA Abx**
		+	**Not Empirically Treat**	**187/957 (19.5%)**	**187 cases may get early MRSA Abx**	
	**False Positive 743 (77.6%)**	-	Empirically Treat	85/957 (8.9%)	NA	
		-	*Not Empirically Treat*	*658/957 (68.8%)*	*658 cases may receive unnecessary MRSA Abx*	
**Low Risk 45,533 NPV: 99.8**	**True Negative 45,425 (99.8%)**	-	**Empirically Treat**	**1,624/45,533 (3.5%)**	**1,624 cases may avoid unnecessary MRSA Abx**	**966 cases may avoid unnecessary MRSA Abx**
		-	Not Empirically Treat	43,801/45,533 (96.2%)	NA	
	**False Negative 108 (0.2%)**	+	*Empirically Treat*	*30/45,533 (0.07%)*	*30 cases may delay MRSA Abx*	
		+	Not Empirically Treat	78/45,533 (0.17%)	NA	

Supplementary Table 5-f: Potential Clinical Impact of LGBM Model Based on Any Antimicrobials Possessing MRSA Activities

MHHS Data (LGBM)						
**Model Predictions**		**MRSA Cx**	**Treating Clinician's decision**	**Cases**	**Potential Benefit and Harm of Model**	**Overall Potential Benefit**
**High Risk 1,370 PPV: 63.6**	**True Positive 875 (63.9%)**	+	Empirically Treat	408/1370 (29.8%)	NA	**440 cases may receive early MRSA Abx**
		+	**Not Empirically Treat**	**464/1370 (33.9%)**	**464 cases may get early MRSA Abx.**	
	**False Positive 498 (36.4%)**	-	Empirically Treat	256/1370 (18.7%)	NA	
		-	*Not Empirically Treat*	*242/1370 (17.8%)*	*242 cases may receive unnecessary MRSA Abx*	
**Low Risk 6,363 NPV:98.5**	**True Negative 6,265 (98.5%)**	-	**Empirically Treat**	**1,305/6,363 (20.5%)**	**1,305 cases may avoid unnecessary MRSA Abx.**	**1063 cases may avoid unnecessary MRSA Abx**
		-	Not Empirically Treat	4,960/6,363 (78.0%)	NA	
	**False Negative 98 (1.5%)**	+	*Empirically Treat*	*24/6,363 (0.37%)*	*24 cases may delay MRSA Abx*	
		+	Not Empirically Treat	74/6,363 (1.2%)	NA	
**MIMIC-IV (LGBM)**						
**Model Predictions**		**MRSA Cx**	**Treating Clinician's decision**	**Cases**	**Potential Benefit and Harm of Model**	**Overall Potential Benefit**
**High Risk 942 PPV: 17.5**	**True Positive 165 (17.5%)**	+	Empirically Treat	18/942 (1.9%)	NA	**126 cases may receive early MRSA Abx**
		+	**Not Empirically Treat**	**147/942 (15.7%)**	**147 cases may get early MRSA Abx**	
	**False Positive 777 (82.5%)**	-	Empirically Treat	69/942 (7.3%)	NA	
		-	*Not Empirically Treat*	*708/942 (76.3%)*	*708 cases may receive unnecessary MRSA Abx*	
**Low Risk 44,437 NPV: 99.8**	**True Negative 48,343 (99.8%)**	-	**Empirically Treat**	**1,384/44,437 (3.1%)**	**1,384 cases may avoid unnecessary MRSA Abx**	**676 cases may avoid unnecessary MRSA Abx**
		-	Not Empirically Treat	42,942/44,437 (96.6%)	NA	
	**False Negative 111 (0.2%)**	+	*Empirically Treat*	*21/44,437 (0.05%)*	*21 cases may delay MRSA Abx*	
		+	Not Empirically Treat	90/44,437 (0.2%)	NA	

Supplementary Table 5-g: Potential Clinical Impact of LR Model Based on Any Antimicrobials Possessing MRSA Activities

MHHS Data (LR)						
**Model Predictions**		**MRSA Cx**	**Treating Clinician's decision**	**Cases**	**Potential Benefit and Harm of Model**	**Overall Potential Benefit**
**High Risk 1,117 PPV:55.7**	**True Positive 1,002 (64.7%)**	+	Empirically Treat	353/1,117 (31.6%)	NA	**253 cases may receive early MRSA Abx**
		**+**	**Not Empirically Treat**	**269/1,117 (24.1%)**	**269 cases may get early MRSA Abx.**	
	**False Positive 547 (35%)**	-	Empirically Treat	353/1,117 (31.6%)	NA	
		-	*Not Empirically Treat*	*194/1.117 (17.4%)*	*194 cases may receive unnecessary MRSA Abx*	
**Low Risk 5,726 NPV:98.3**	**True Negative 5,628 (98.3%)**	**-**	**Empirically Treat**	**961/5,726 (14.4%)**	**961 cases may avoid unnecessary MRSA Abx.**	**767 cases may avoid unnecessary MRSA Abx**
		-	Not Empirically Treat	4,667/5,726 (81.5%)	NA	
	**False Negative 98 (0.7%)**	+	*Empirically Treat*	*16/5,726 (0.3%)*	*16 cases may delay MRSA Abx*	
		+	Not Empirically Treat	82/5,726 (1.4%)	NA	
**MIMIC-IV (LR)**						
**Model Predictions**		**MRSA Cx**	**Treating Clinician's decision**	**Cases**	**Potential Benefit and Harm of Model**	**Overall Potential Benefit**
**High Risk 2,187 PPV:22.4**	**True Positive 261 (11.9%)**	+	Empirically Treat	36/2,187 (1.6%)	NA	**202 cases may receive early MRSA Abx**
		**+**	**Not Empirically Treat**	**225/2,187 (10.4%)**	**225 cases may get early MRSA Abx**	
	**False Positive 1,926 (88.0%)**	-	Empirically Treat	214/2,187 (8.1%)	NA	
		-	*Not Empirically Treat*	*1,712/2,187 (80.0%)*	*,712 cases may receive unnecessary MRSA Ab*	
**Low Risk 41,887 NPV:99.7**	**True Negative 41,775 (99.7%)**	**-**	**Empirically Treat**	**1,301/41,887 (3.1%)**	**1,301 cases may avoid unnecessary MRSA Abx**	*411 cases may receive unnecessary MRSA Abx*
		-	Not Empirically Treat	40,474/41,887 (96.6%)	NA	
	**False Negative 112 (0.3%)**	+	*Empirically Treat*	*23/41,887 (0.05%)*	*23 cases may delay MRSA Abx*	
		+	Not Empirically Treat	89/41,887 (0.2%)	NA	

Supplementary Figure 10. AUROC Changes for Repeated Index Events

Supplementary Figure 10-a: AUROC Change in MHHS dataset

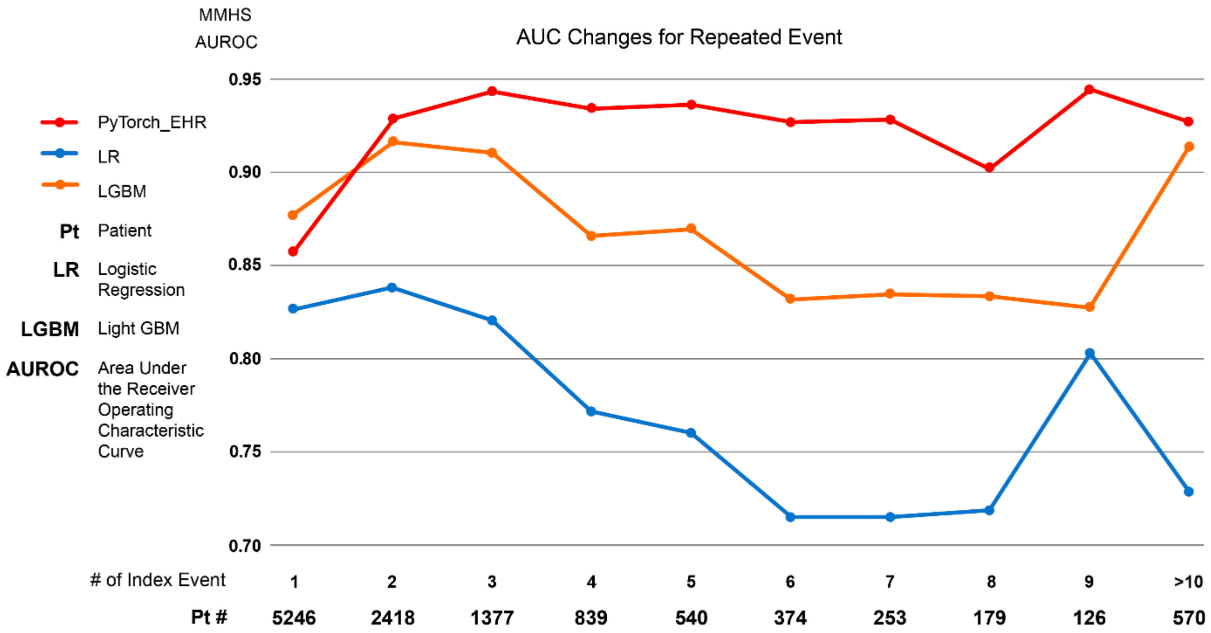

Supplementary Figure 10-b: AUROC Change in MIMIC-IV dataset

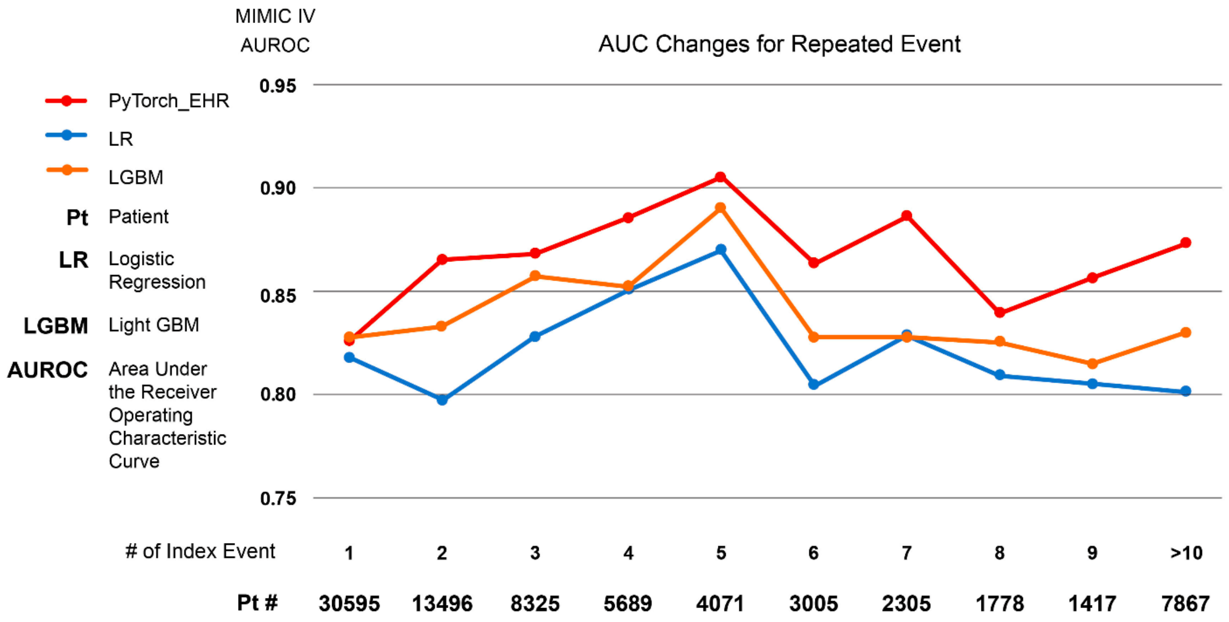

This figure summarizes the changes in Area Under Receiver Operating Characteristic Curve (AUROC) at the repeated index event. For example, 2 on the x-axis means AUROC only for patients who had the 2nd index event in the test datasets. As indicated in this figure, PyTorch_EHR improved the AUROC when the patient had repeated events. Patients with multiple index points had a longer sequence of HER data compared to patients with the first index time.
