# Supplementary material for: Deep learning model for personalized prediction of positive MRSA culture using time-series electronic health records
Source: Nat Commun. 2024 Mar 6;15:2036. doi: 10.1038/s41467-024-46211-0 (PMC10917736; doi:10.1038/s41467-024-46211-0)
Supplement: Supplementary file 3 — Reporting Summary [file 41467_2024_46211_MOESM3_ESM.pdf]

nature portfolio

Corresponding author(s): Masayuki Nigo

Last updated by author(s): Feb 5, 2024

Reporting Summary

Nature Portfolio wishes to improve the reproducibility of the work that we publish. This form provides structure for consistency and transparency in reporting. For further information on Nature Portfolio policies, see our Editorial Policies and the Editorial Policy Checklist.

Statistics

For all statistical analyses, confirm that the following items are present in the figure legend, table legend, main text, or Methods section.

n/a	Confirmed	
☐	☒	The exact sample size (*n*) for each experimental group/condition, given as a discrete number and unit of measurement
☐	☒	A statement on whether measurements were taken from distinct samples or whether the same sample was measured repeatedly
☐	☒	The statistical test(s) used AND whether they are one- or two-sided<br>*Only common tests should be described solely by name; describe more complex techniques in the Methods section.*
☐	☒	A description of all covariates tested
☒	☐	A description of any assumptions or corrections, such as tests of normality and adjustment for multiple comparisons
☐	☒	A full description of the statistical parameters including central tendency (e.g. means) or other basic estimates (e.g. regression coefficient) AND variation (e.g. standard deviation) or associated estimates of uncertainty (e.g. confidence intervals)
☐	☒	For null hypothesis testing, the test statistic (e.g. *F*, *t*, *r*) with confidence intervals, effect sizes, degrees of freedom and *P* value noted<br>*Give P values as exact values whenever suitable.*
☒	☐	For Bayesian analysis, information on the choice of priors and Markov chain Monte Carlo settings
☒	☐	For hierarchical and complex designs, identification of the appropriate level for tests and full reporting of outcomes
☒	☐	Estimates of effect sizes (e.g. Cohen's *d*, Pearson's *r*), indicating how they were calculated

Our web collection on statistics for biologists contains articles on many of the points above.

Software and code

Policy information about availability of computer code

Data collection	Our sample codes for pre-processing and model training are avabilable at https://github.com/ZhiGroup/pytorch_ehr/tree/MRSA.
Data analysis	Python Ver 3.9.7, Optuna ver. 2.10.0, PyTorch ver. 1.7.1 and Sklearn ver. 0.24.2

For manuscripts utilizing custom algorithms or software that are central to the research but not yet described in published literature, software must be made available to editors and reviewers. We strongly encourage code deposition in a community repository (e.g. GitHub). See the Nature Portfolio guidelines for submitting code & software for further information.

Data

Policy information about availability of data

All manuscripts must include a data availability statement. This statement should provide the following information, where applicable:

- Accession codes, unique identifiers, or web links for publicly available datasets
- A description of any restrictions on data availability
- For clinical datasets or third party data, please ensure that the statement adheres to our policy

MIMIC-IV data v2.1 is publicly available after data use agreement on the website. (https://physionet.org/content/mimiciv/2.1/) Analysis in the Memorial Hermann Hospital System was conducted under a waiver of informed consent with approval from the University of Texas Health Science Center at Houston (UTHSCH) and Memorial Hermann Hospital System (MHHS). MHHS data that support the findings of this study are not openly available due to reasons of sensitivity and are

available from the corresponding author upon reasonable requests. MIMIC-IV data v2.1 is publicly available after data use agreement on the website. (https://physionet.org/content/mimiciv/2.1/)

Research involving human participants, their data, or biological material

Policy information about studies with human participants or human data. See also policy information about sex, gender (identity/presentation), and sexual orientation and race, ethnicity and racism.

Reporting on sex and gender	We only used "gender" in our study. The information was obtained via self-reported fashion.
Reporting on race, ethnicity, or other socially relevant groupings	We used race and ethnicity to characterize the patient population in Memorial Hermann Hospital System and MIMIC-IV datasets. We did not use those to characterize the social-economic conditions.
Population characteristics	In characterizing our patient population within the datasets, we included a range of variables: age, gender, ethnicity, race, and primary language, alongside past medical diagnoses, types of treatments received, and types of tests conducted. These variables were selected not only for their relevance in describing the demographic and clinical profile of the patients but also due to their potential predictive value in identifying MRSA culture positivity.
Recruitment	Patients were randomly recruited from database in Memorial Hermann Hospital Dataset. For MIMIC-IV, all available patients who had at least one microbiology culture were included in the study.
Ethics oversight	The University of Texas Health Science Center at Houston and Memorial Hermann Hospital System (Protocol number: HSC-MS-20-0121)

Note that full information on the approval of the study protocol must also be provided in the manuscript.

Field-specific reporting

Please select the one below that is the best fit for your research. If you are not sure, read the appropriate sections before making your selection.

☒ Life sciences ☐ Behavioural & social sciences ☐ Ecological, evolutionary & environmental sciences

For a reference copy of the document with all sections, see nature.com/documents/nr-reporting-summary-flat.pdf

Life sciences study design

All studies must disclose on these points even when the disclosure is negative.

Sample size	We did not perform a formal sample size calculation. For the MHHS database, our sample comprised the number of MRSA patients identified during the study period. We employed a 1:3 ratio for control to maintain balanced datasets. For the MIMIC-IV dataset, we included all patients who met our inclusion criteria. The rationale for these choices is based on the premise that deep learning projects typically yield more robust predictions with larger patient cohorts.
Data exclusions	Patients who were less than 18 years old.
Replication	Our experiments were replicated multiple times to ensure our findings. Each experimental procedure was performed independently on different occasions, confirming the consistency of our results. All attempts at replication were successful, demonstrating the reproducibility of our study using the MIMIC-IV dataset. This dataset, being publicly available and widely used in clinical research, adds to the reliability of our replication efforts.
Randomization	This study is retrospective in nature; therefore, randomization was not applicable. Our analysis relied on existing data from the MHHS and MIMIC-IV databases, where patient selection was based on predefined inclusion criteria rather than random assignment.
Blinding	In our retrospective study, blinding was implemented in the division of datasets. Patients were randomly split into training, validation, and test datasets based on their unique identifiers. This randomization process ensured that the allocation to each dataset was unbiased, aligning with common practices in deep learning research. We utilized a 70:10:20 ratio for dividing the datasets, as this distribution is widely accepted and provides a balanced approach for training and evaluating the model's performance.

Reporting for specific materials, systems and methods

We require information from authors about some types of materials, experimental systems and methods used in many studies. Here, indicate whether each material, system or method listed is relevant to your study. If you are not sure if a list item applies to your research, read the appropriate section before selecting a response.

Materials & experimental systems

n/a	Involved in the study
☒	☐ Antibodies
☒	☐ Eukaryotic cell lines
☒	☐ Palaeontology and archaeology
☒	☐ Animals and other organisms
☒	☐ Clinical data
☒	☐ Dual use research of concern
☒	☐ Plants

Methods

n/a	Involved in the study
☒	☐ ChIP-seq
☒	☐ Flow cytometry
☒	☐ MRI-based neuroimaging
